# Development and Analysis of Bilayer Foamed Oleogels Stabilized with Ecogel™: Exploring the Role of Tween 80 in Modifying Physicochemical Properties

**DOI:** 10.3390/ijms252312632

**Published:** 2024-11-25

**Authors:** Sonia Kudłacik-Kramarczyk, Anna Drabczyk, Alicja Przybyłowicz, Weronika Kieres, Marcel Krzan

**Affiliations:** 1 Jerzy Haber Institute of Catalysis and Surface Chemistry, Polish Academy of Sciences, 8 Niezapominajek St., 30-239 Krakow, Poland; alicja.przybylowicz@student.pk.edu.pl (A.P.); weronikakieres1605@gmail.com (W.K.); marcel.krzan@ikifp.edu.pl (M.K.); 2 CBRTP SA—Research and Development Center of Technology for Industry, Ludwika Waryńskiego 3A St., 00-645 Warsaw, Poland; 3 Faculty of Mechanical Engineering, Cracow University of Technology, 37 Jana Pawła II Av., 31-864 Krakow, Poland

**Keywords:** structured emulsions, hydrophobicity modulation, surface wettability, physicochemical properties, rheology, polysorbate

## Abstract

Oleogels are structured materials formed by immobilizing oil within a polymer network. This study aimed to synthesize bilayer foamed oleogels using Ecogel™ as an emulsifier—a natural gelling and emulsifying agent commonly used to stabilize emulsions. Ecogel™ is multifunctional, particularly in cosmetic formulations, where it aids in creating lightweight cream gels with a cooling effect. However, the specific goal of this study was to investigate the physicochemical properties of oleogels formed with Ecogel™, Tween 80, gelatin, and glycerin. The combination of these ingredients has not been studied before, particularly in the context of bilayer foamed oleogels. The biphasic nature of the resulting materials was explored, consisting of a uniform lower phase and a foamed upper layer. Several analytical techniques were employed, including FT-IR spectrophotometric analysis, moisture content evaluation, surface wettability measurements, microscopic imaging, and rheological studies, in addition to surface energy determination. The results demonstrated that the addition of Tween 80 significantly improved the stability and rigidity of the oleogels. Furthermore, storage at reduced temperatures after synthesis enhanced the material’s stabilizing properties. These materials also showed an affinity for interacting with non-polar compounds, indicating potential applications in skincare, especially for interaction with skin lipids.

## 1. Introduction

An oleogel is a gel-like structure formed by immobilizing oil in a polymer network, providing the desired consistency. In the pharmaceutical and cosmetic industries, oleogels are gaining attention as carriers of active substances due to their structural properties and ability to incorporate hydrophobic compounds [1,2]. Oleogels are typically oil-based gels, formed using oils such as liquid paraffin or fatty oils, which are thickened using colloidal silica, or soaps such as aluminum or zinc stearates. One of the substances commonly used to obtain oleogels is gelatin. It is a thermo-reversible protein hydrocolloid extracted from animals, mainly from the hides and bones of cattle [3,4], that is formed through the hydrolysis of collagen [5,6]. Gelatin is characterized by a small difference between its melting and gelling points, providing unique functional properties. It is widely used as a stabilizing, thickening, gelling, and film-forming agent, as well as an emulsifier [3,7,8]. It also prevents delamination, ensuring proper consistency, flexibility, and form retention (e.g., in tablets) [9]. Additionally, gelatin binds water without affecting the smell or taste of products, making it ideal for food, pharmaceutical, and cosmetic applications [10,11]. It is also used in the production of adhesives and to create biological models in 3D printing [12].

To modify the rheological properties and improve the structural integrity of oleogels, components like glycerin are often added to the gel matrix or oil phase [13]. Glycerin is a colorless, odorless alcohol with strong water-binding properties, making it an excellent moisturizing agent [14,15]. It dissolves well in water, has a slightly sweet taste, and is calorie-free and non-toxic, making it a suitable sugar substitute [16,17]. In the food industry, it prevents drying, extends the shelf life of products, and is a good stabilizer. In the pharmaceutical industry, it is the base for syrups [18,19] and is used in dermatological cosmetics in the form of creams, lotions, and ointments [14]. Another key modifying agent is Ecogel™, a natural gelling and emulsifying agent that stabilizes emulsions and allows for the creation of lightweight cream gels with a cooling effect [20]. Ecogel™ is widely used in cosmetics and the production of edible, biodegradable coatings for fruits, protecting them from drying and mechanical damage and extending the shelf life of sliced fruits [21].

Emulsifiers are essential in oleogel formulations, helping stabilize emulsions and enhance component dispersion. One of the most commonly used emulsifiers in oleogel synthesis is Tween 80, a non-ionic surfactant made from fatty acid esters and polyoxyethylene sorbitan monooleate. Despite the extensive use of emulsifiers like Tween 80 in various formulations, its role in stabilizing bilayer foamed oleogels remains underexplored. This study uniquely combined Tween 80 with Ecogel™, gelatin, and glycerin to create oleogels with enhanced physicochemical properties. The biphasic structure, consisting of a foamed upper layer and a uniform lower layer, introduces new possibilities for tailoring material properties such as wettability and stability. This approach provides a novel platform for dermal applications, addressing current challenges in the pharmaceutical and cosmetic industries, including improved bioavailability and moisture retention in sensitive skin formulations [22,23]. Due to its distinctly hydrophilic structure, it acts as an O/W (oil-in-water) emulsifier where the dispersing phase is water and the dispersed phase is oil, which ensures the stability of emulsion systems and the effective homogenization of components [24,25]. In the food industry, it is used to stabilize products containing water and fats, such as sauces [26]. In pharmaceuticals, it functions as a carrier of active substances or a stabilizing agent [27]. In cosmetics, it combines water and oil phases, improving texture and creating a uniform formula, while, in shampoos, for example, it is responsible for foaming properties [28].

This study offers a novel approach to oleogel formulation by combining Ecogel™, Tween 80, gelatin, and glycerin, resulting in bilayer foamed oleogels with enhanced physicochemical properties. Unlike traditional oleogels, the inclusion of these components facilitates the creation of structures with improved wettability, rheological characteristics, and stability, while also allowing for lightweight textures suitable for dermal applications. These unique features make bilayer foamed oleogels particularly attractive for use as carriers of active substances in pharmaceutical and cosmetic formulations. By stabilizing emulsions and enhancing moisture retention, these oleogels hold the potential for diverse applications, including in wound care, anti-aging formulations, and dermocosmetics for sensitive skin.

The potential practical applications of bilayer foamed oleogels lie in their ability to serve as multifunctional carriers in cosmetic and pharmaceutical formulations. These oleogels can improve the stability, bioavailability, and controlled release of active ingredients, making them suitable for advanced skincare formulations, including anti-aging products, wound-healing preparations, and moisturizers for sensitive skin. Furthermore, the lightweight and hydrophilic nature of these structures positions them as ideal candidates for creating innovative, user-friendly dermocosmetic products with enhanced sensory properties.

While research has extensively covered oleogels in food and other industries, the combination of gelatin, glycerin, Ecogel™, and Tween 80 in the context of bilayer foamed oleogels remains unexplored. This study investigated the physicochemical properties of oleogels prepared using these components, including their rheological properties, wettability, sorptive properties, and stability. Additionally, the chemical structure of the oleogels was analyzed via FT-IR spectroscopy and their morphology was assessed using optical microscopy. This research contributes to the understanding of oleogels’ potential in pharmaceutical and cosmetic applications, particularly for use in dermal formulations.

## 2. Results

### 2.1. Analysis of Oleogels via FT-IR Spectroscopy

**Figure 1:**
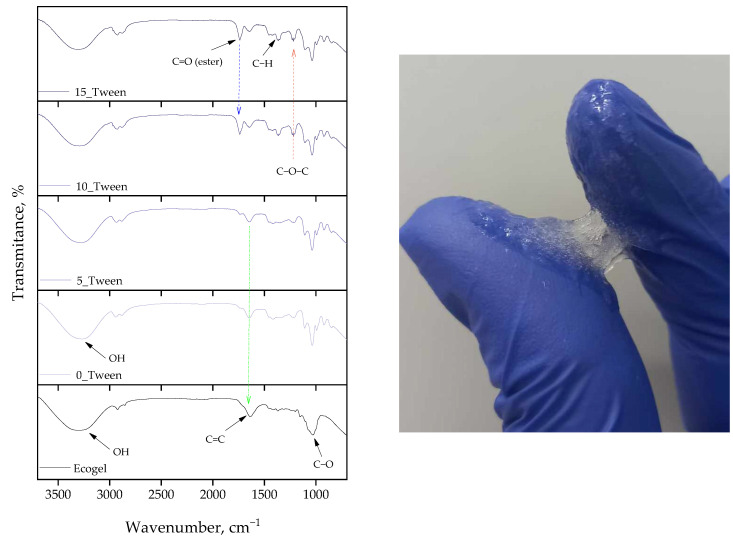
FT-IR spectra of all tested samples, both oleogels and Ecogel™. The experiment was conducted in triplicate and the presented data represent a representative result from the measurements.

**Figure 2:**
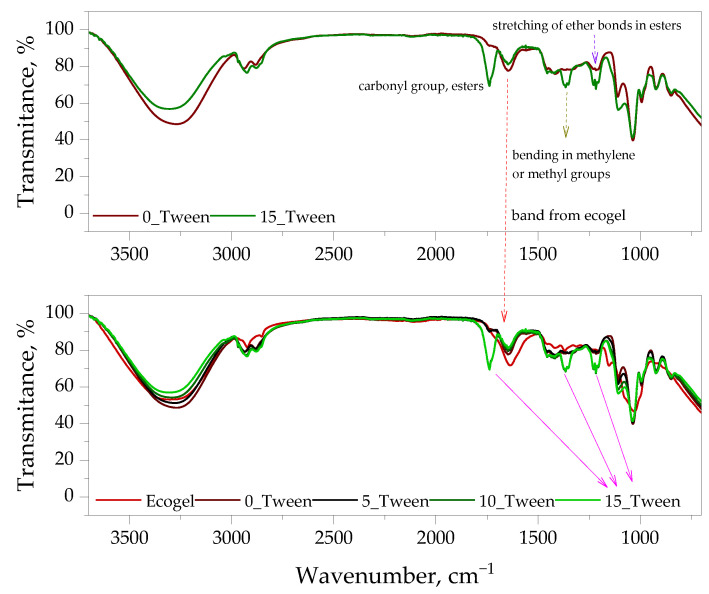
Compilation of FT-IR spectra of oleogel sample without Tween and with the highest content of this emulsifier (**up**) and compilation of FT-IR spectra of all test samples (**down**). The experiment was conducted in triplicate and the presented data represent a representative result from the measurements.

All obtained oleogel samples were subjected to FT-IR spectroscopic analysis. In addition, analysis was also performed for Ecogel™. Obtained FT-IR spectra are presented below in Figure 1 and Figure 2.

FT-IR analysis allowed us to characterize the chemical structure of the oleogels and identify possible interactions between their components, including emulsifiers. Notably, the addition of Tween 80 resulted in the appearance of additional peaks corresponding to carbonyl groups, indicating enhanced stability of the oleogel structure.

### 2.2. Microscopic Assessment of Oleogels’ Morphology

**Figure 3:**
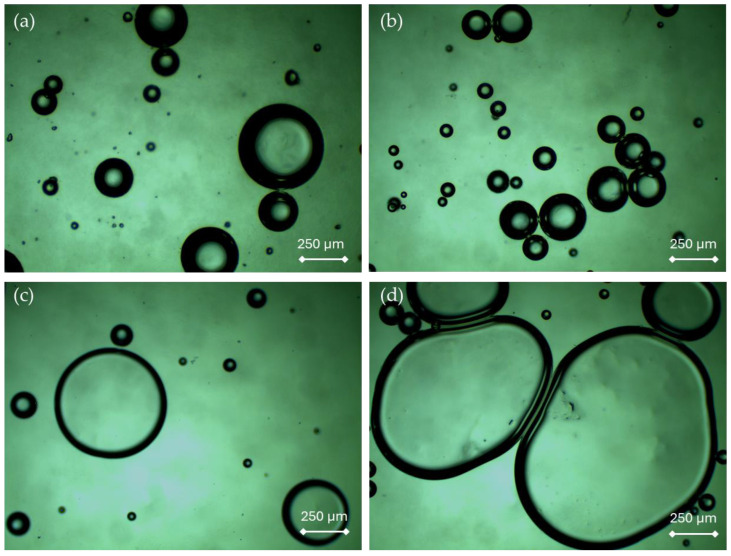
Images of oleogel samples: 0_Tween (**a**), 5_Tween (**b**), 10_Tween (**c**), and 15_Tween (**d**), magnifications: 4×. The experiment was conducted in triplicate and the presented data represent a representative result from the measurements.

Below in Figure 3, images of the prepared oleogel samples are presented.

Imaging of oleogels by means of an optical microscope was performed to determine the effect of oleogels’ composition on their morphology and structural homogeneity. This study allowed for a visual comparison of the obtained samples as well as the identification of possible differences resulting from the use of different contents of emulsifiers.

### 2.3. Evaluation of Oleogels’ Surface Wettability

**Figure 4:**
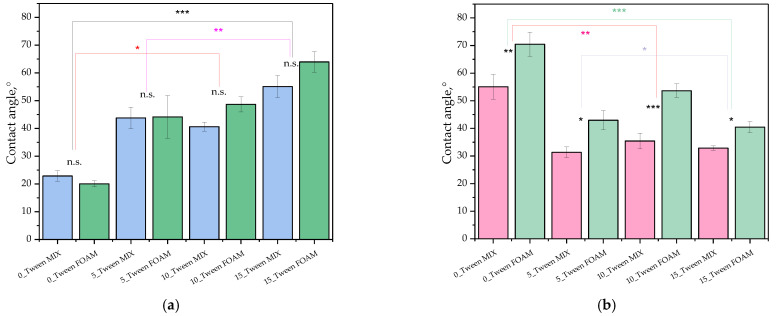
Contact angles of oleogel samples (both mixtures and foams). Panel (**a**) represents measurements for water and panel (**b**) represents measurements for diiodomethane. Error bars represent standard deviations (*n* = 3). Statistical significance (*p*-values) between MIX and FOAM for each concentration is indicated above the bars (n.s. = not significant, * *p* < 0.05, ** *p* < 0.01, for *** *p* < 0.001). Significant differences between concentrations (Tukey’s HSD) are marked with asterisks above the corresponding groups.

**Figure 5:**
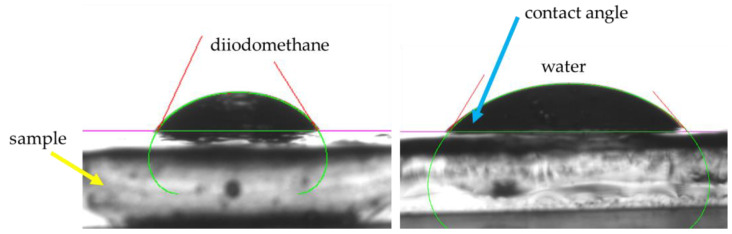
The schematic behavior of both hydrophobic (diiodomethane) and hydrophilic (pure double-distilled water) liquid in contact with test samples.

**Figure 6:**
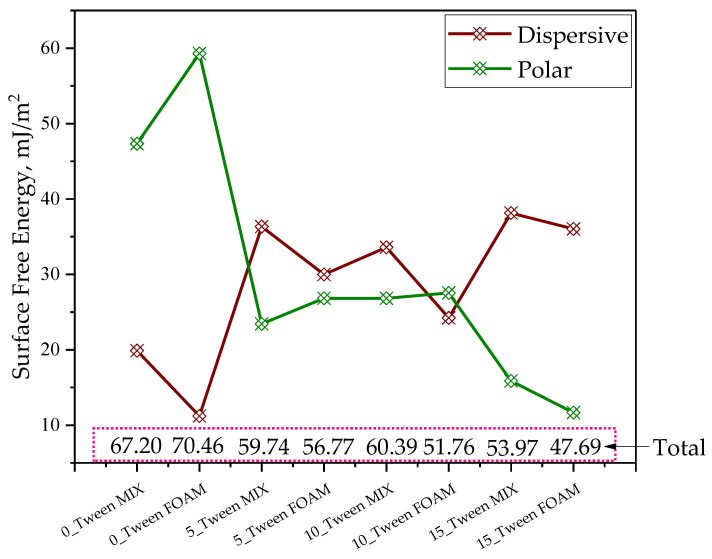
Results of surface free energy and its components (dispersive and polar energy) for each oleogel sample (two forms: mixture (noted as MIX and FOAM). The values presented were calculated based on the averaged contact angle measurements from Figure 4. Statistical analysis was not performed due to a lack of repeated measurements for surface energy components.

As part of the experiments, studies on the oleogels’ wettability were also conducted. In the case of each sample, studies were carried out both for its homogenized mixture as well as for its foam (forming on the sample’s surface before oleogel mixing). Below, the values of the determined contact angles for each sample are presented (Figure 4) as well as a scheme of the behavior of various liquids (both hydrophilic and hydrophobic) in contact with the test samples (Figure 5). Next, Figure 6 demonstrates the values of surface free energy calculated for the oleogel samples as well as its dispersive and polar components.

Studies on oleogels’ wettability make it possible to determine their hydrophilic–hydrophobic nature, which is key to understanding their potential applications, such as for drug carriers or adhesion agents. Measurements of surface free energy, on the other hand, enable the determination of the surface characters of samples, which is important for predicting their interactions with other materials.

### 2.4. Analysis of Sorption Behavior of Oleogels Under Controlled Humidity Conditions

**Figure 7:**
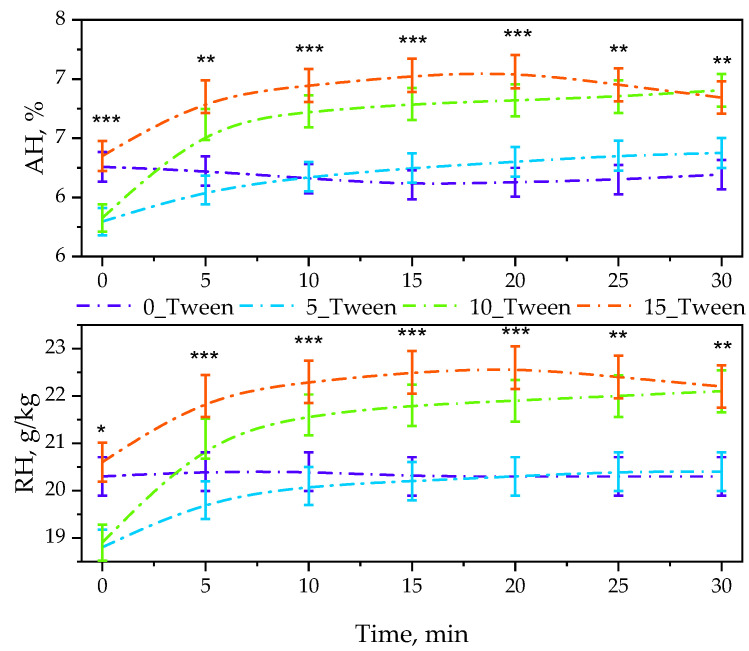
Determined values of the absolute humidity (AH, up) and relative humidity (RH, down) of the oleogels. Error bars represent standard deviations (*n* = 3). Statistical significance between groups was analyzed using one-way ANOVA for each time point. Significant differences between groups are indicated with asterisks: *p* < 0.05 (*), *p* < 0.01 (**), *p* < 0.001 (***).

During the performed research, studies to determine the oleogels’ sorption properties were also conducted. Their results in the form of both absolute humidity (AH) and relative humidity (RH) for each oleogel are compiled below in Figure 7.

Studies on the sorptive properties of oleogels aim to evaluate a material’s capability to absorb and retain moisture from the environment, which can have a significant impact on its stability and interactions with the environment. Importantly, these investigations make it possible to determine how oleogels will react to changes in environmental humidity, which, in turn, is important in terms of their potential applications, e.g., as carriers of active substances.

### 2.5. Stability Assessment of Oleogels Using Multiscan System

**Figure 8:**
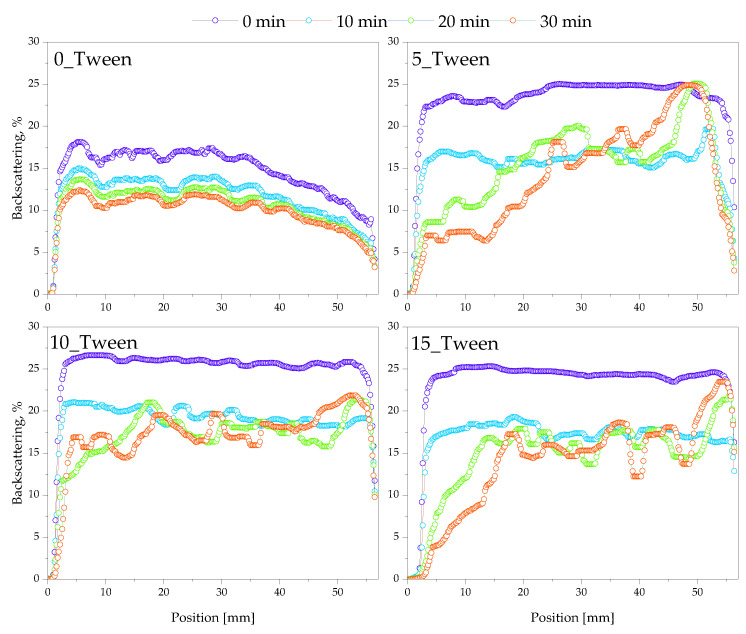
Results of the stability analysis of the oleogels directly after their synthesis (30 min measurement). The experiment was conducted in triplicate and the presented data represent a representative result from the measurements.

**Figure 9:**
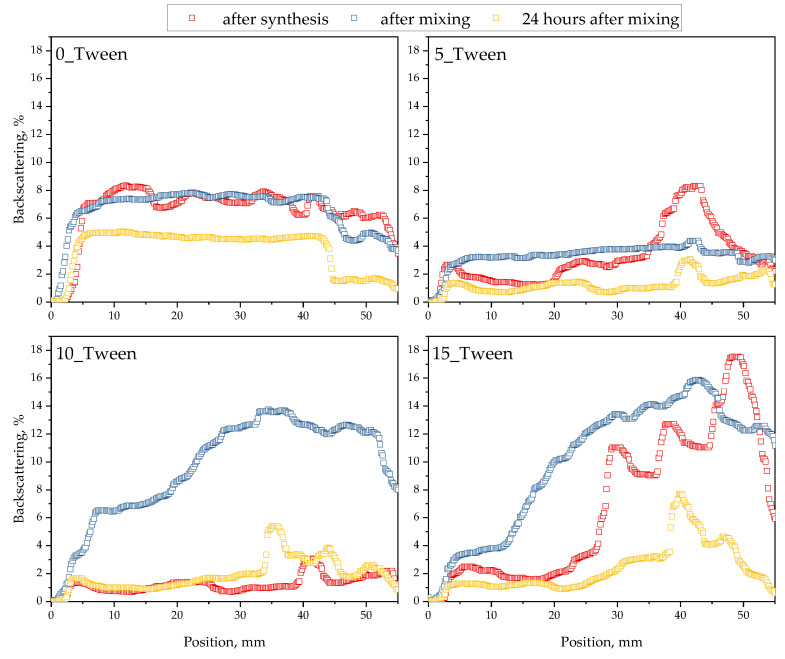
Compilation of all stability measurements of the oleogels (directly after the synthesis, after mixing, and 24 h after mixing and storage at 6 °C). The experiment was conducted in triplicate and the presented data represent a representative result from the measurements.

**Figure 10:**
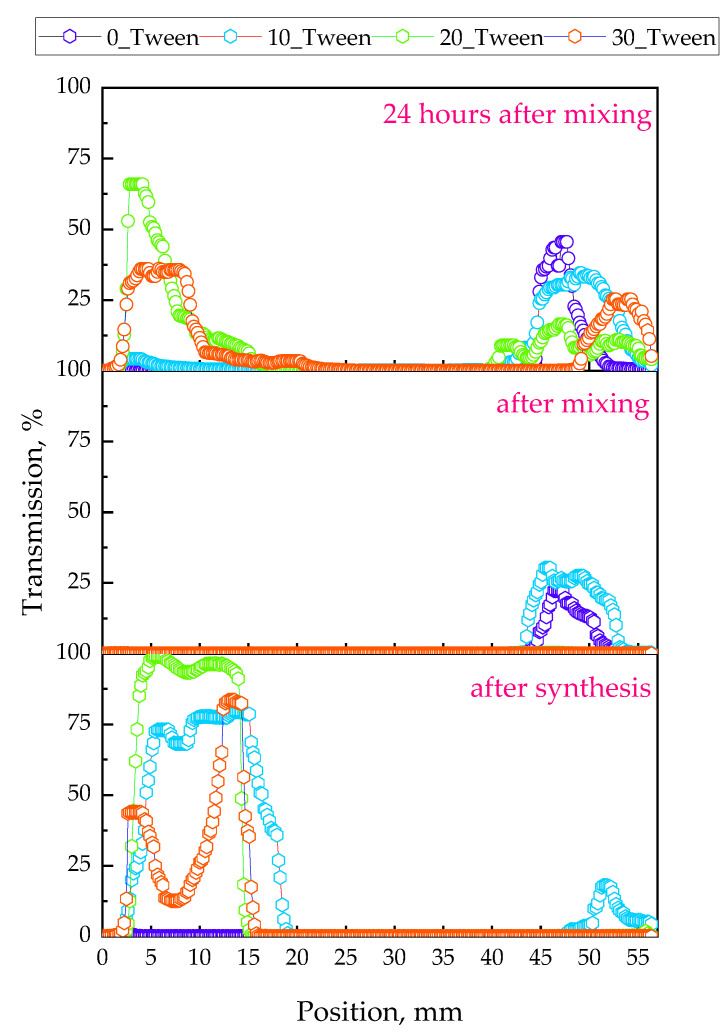
Compilation of the transmission measurements for the oleogel samples (directly after the synthesis, after mixing, and 24 h after mixing and storage at 6 °C). The experiment was conducted in triplicate and the presented data represent a representative result from the measurements.

The results of the evaluation of the oleogels’ stability are demonstrated below in Figure 8, Figure 9 and Figure 10.

Analysis of the oleogels’ stability via the multiscan system was performed to verify their homogeneity and structural stability over time. This made it possible to detect possible phase separation or other phenomena like sedimentation or destabilization processes in the oleogels, which is crucial in the context of considering their suitability for various applications.

### 2.6. Analysis of Oleogels’ Rheology

**Figure 11:**
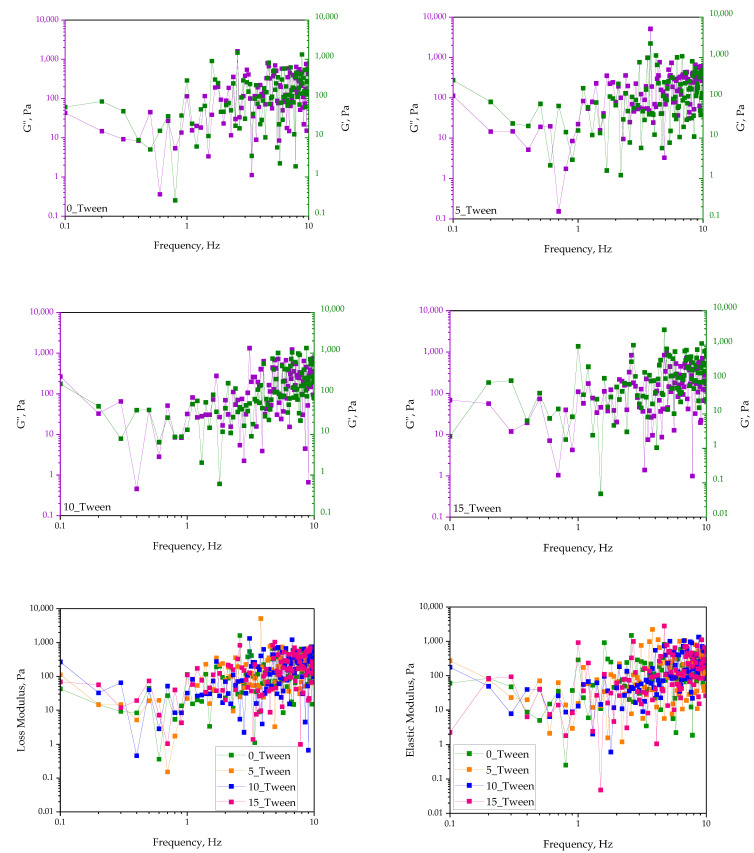
Evolution of the elastic and loss moduli in the oleogels with the frequency. The experiment was conducted in triplicate and the presented data represent a representative result from the measurements.

The results of the rheology analysis are presented below in Figure 11.

The experiment also involved theological investigations. The main objective of these tests was to determine the viscosity and elastic properties of the oleogels, which allowed us to determine their susceptibility to deformation under external forces.

## 3. Discussion

The first of the studies conducted on the analyzed materials was the FT-IR analysis. In this study, the materials were tested in the form of a mix. In Figure 1, we observe a photograph showing the consistency of the material obtained using the highest amount of Tween. In the same figure, FTIR spectra are presented, comparing all the tested samples. Additionally, the spectrum for the Ecogel™ itself is shown, which, as we can observe, exhibited characteristic bands in the range of 3200–3500 cm^−1^ (O-H), 1600 cm^−1^ (aromatic C = C), and 1020 cm^−1^ (C-O). This spectrum was essential for observing differences in the spectra of the obtained materials, where we could see the emergence of all the aforementioned bands from the Ecogel™ in the structure of the obtained samples. Interestingly, it was only for the material containing an additional 33% and 50% of Tween compared to glycerol that we observed the appearance of additional bands in the areas of 1700 cm^−1^ (C = O, carbonyl group, esters), 1360 cm^−1^ (C-H, bending in methylene or methyl groups), and 1210 cm^−1^ (C-O-C, stretching of ether bonds in esters), which was also confirmed by Liang and Zhu in their article [29]. This aligns with assumptions stating that a greater amount of a given additive allows for more intense bands from that substrate to be observed in the spectra [30]. To better illustrate these differences, it was decided to overlay the appropriate spectra to observe these differences even more distinctly; thus, in Figure 2, we can see the aforementioned bands, which stand out prominently against the other spectra. The greatest intensity of the new bands was observed with additions of 33% and 50% Tween. This analysis did not confirm the formation of any additional bands that could indicate the emergence of toxic by-products or any features suggesting material degradation. This analysis was crucial to confirm the proportions used in the ingredients during the synthesis stage and served as an additional verification necessary for a precise analysis of the obtained results. Moreover, in the spectra, we observed bands from the other components included in the obtained materials, such as glycerol (the characteristic signal for the C-O bond could be found in the range of 1000–1150 cm^−1^) and gelatin (amide I, II, and III), which other researchers also demonstrated in their works, i.e., Yoda and Ootawa [31]. These groups were not marked in the spectra because their quantity in each sample was constant; therefore, it was not significant in the context of this work.

Next, surface morphology analysis was conducted using an optical microscope (Figure 3), where it was observed that the addition of Tween causes an increase in the diameter of the bubbles observed in the obtained microphotographs, where a similar effect is also indicated by Górecki and Antenucci [32]. According to Stortz and Marangoni, such an increase in bubble size can improve the spreadability and skin absorption properties of oleogels, suggesting potential advantages for cosmetic applications [33]. Since Tween is a compound that lowers surface tension [34] between phases, its addition may result in the formation of larger, more stable bubbles that do not burst. Thus, it can be inferred that larger bubbles may remain stable for a longer time. The foamy structure of these materials resulted from the introduction of air into the system during very intense mixing using a homogenizer. To deepen this analysis, rheological analysis and analysis using multiscanning were also conducted, which answered emerging questions. The discussion regarding this aspect can be found later in this chapter.

Before proceeding to that, let us first discuss the results concerning surface wetting, which was also significantly related to the structure of the examined materials. It should be emphasized that this study analyzed each sample from two layers, i.e., from the upper layer (FOAM) and after mixing both layers, which was denoted as MIX. The results of this analysis are presented in Figure 4. This study was conducted in the presence of two solvents, namely, distilled water and diiodomethane, to better understand the obtained results. Figure 5 shows the shape of water or diiodomethane droplets in contact with the tested material. At first glance, we can see clear differences, even though this photograph shows the contact of these two solvents with the same sample. The calculated wetting angles for the tested materials are presented in Figure 4. Additionally, a statistical analysis was conducted for this study to verify statistical significance, and the obtained results for each sample, whether MIX or FOAM, did not show statistical significance. Moving on to a detailed discussion of the results, we could observe that the wetting angles for diiodomethane were significantly larger in the case of the sample not containing Tween, regardless of whether it was MIX or FOAM, whereas, with increasing additions of the discussed surfactant, we could observe the opposite effect, i.e., with the largest amount of Tween, we observed significantly larger wetting angles for the samples in contact with water, indicating the hydrophobic effect of Tween. Furthermore, the foam sample had a greater wetting angle than the mix of this sample, which may have arisen firstly from the structure of the foam, which maintained droplets in an unchanged position through its porous structure, and, secondly, because the foam may have caused greater hydrophobicity of the structure. O’Sullivan et al. found that increased hydrophobicity, as observed in samples with a higher Tween content, supports better interaction with non-polar components, such as skin lipids, further suggesting these oleogels’ suitability for dermal applications [35]. In this case, we can state that samples containing a larger amount of Tween were better wetted by diiodomethane. Based on the obtained results, an analysis of surface energies was also conducted, and the results for the dispersive and polar energies for each sample are presented in Figure 6. The results obtained in this chart suggest that for samples containing Tween, in every case, the dispersive energy was higher than the polar energy, whereas the sample without Tween showed a very high value of polar energy, both for the mixed form and for the foam, which may suggest that this sample had a greater ability to interact with polar components. However, if we consider the values of total energy, those for samples containing Tween were lower than for the sample that did not contain Tween, and, furthermore, mixed samples mostly had lower total energy, which suggests that Tween reduced the surface energy of the material. In summary, we can see that the addition of Tween decreased the potential for interaction of the obtained materials with polar substances, which, from the perspective of dermal applications, is a desirable aspect since samples without Tween have a greater ability to interact with polar skin components, e.g., sweat and moisture, and such a material can thus wet it better; meanwhile, samples with Tween exhibited a more nonpolar character; thus, they would interact better with skin components such as lipids [36].

Another study related to the previous one was the sorption study, also known as the moisture measurement. The results from this study are presented in Figure 7, which shows the measured value of humidity (RH, %) and the calculated value of absolute humidity (AH) based on it. The overall conclusion is that all samples containing Tween demonstrated an increase in humidity in the tested environment in contact with them, unlike the sample not containing Tween, where a constant level of humidity was observed. In the graph illustrating RH changes over time, we can observe that the sample with the largest amount of Tween caused the greatest increase in humidity in the environment, which can be linked to the results concerning the previous study, where samples containing Tween exhibited a more nonpolar character, leading us to conclude that the water present in the sample began to evaporate, hence the observed increase in humidity in the environment. Tween did not favor the binding of water within the sample, which facilitated its release into the environment. In the sample that did not contain Tween, which exhibited a polar character, we can infer that this material had a greater ability to retain water, making it less prone to evaporation than materials containing Tween.

**Figure 12:**
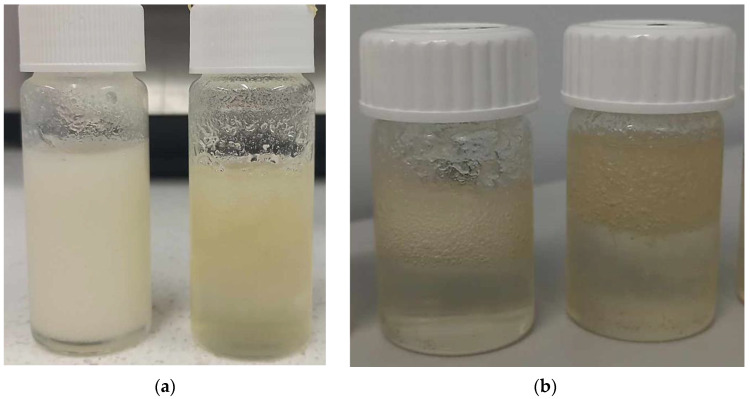
Images of exemplary oleogel samples directly after synthesis (for measurement I via multiscan system) (**a**) and after 24 h of storage at 6 °C (for measurement III via multiscan system) (**b**).

**Figure 13:**
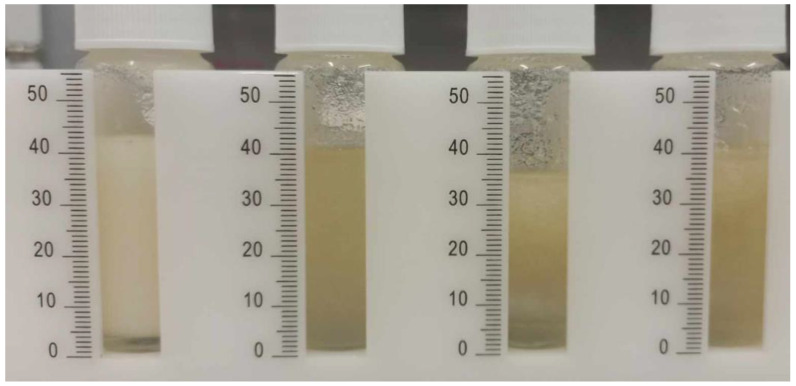
Images of the obtained oleogels. Sequence of samples from the left: 0_Tween, 5_Tween, 10_Tween, 15_Tween.

After conducting an analysis of the surface characteristics of the materials and their interactions with polar and nonpolar liquids, the next stage of this research was to perform an analysis using multiscan technology. Multiscan technology has been widely applied in studies to evaluate stability and phase separation in emulsions and similar dispersions [37,38]. Figure 8 presents a comparison of backscattering for four examined samples during continuous scanning for 30 min, with a scanning interval of 10 min. Analyzing the obtained results, i.e., backscattering for the tested materials directly after synthesis, we can observe that the sample not containing Tween had the lowest values of backscattering, which decreased over the 30 min. This likely indicates a more homogeneous structure of this sample compared to the others, resulting in less light scattering than the samples with Tween. As noted in similar studies, lower backscattering values often correlate with greater homogeneity and phase stability [39]. However, it should also be emphasized that at the initial position, the level of backscattering was higher than at the final position of the scan, which may indicate phase separation or less stability compared to the samples containing Tween. For the samples containing Tween, at time zero, all samples exhibited the highest backscattering at around 25%, at a uniform level across the entire position of the sample, suggesting a non-homogeneous structure of the material and, thus, greater scattering. Tween is known to stabilize bubbles within emulsions, which increases light scattering and often contributes to the structured heterogeneity of the samples [40]. The large number of bubbles throughout the volume of the sample caused light scattering throughout the volume [41]. However, after some time, there was a phenomenon where backscattering was initially lower, averaging around 10%, but, at the final position, it reached 25%, equal to the level measured at time zero. This suggests the separation of the mixture into two phases: a lower phase, which was more homogeneous and scattered less radiation, and an upper phase, which formed in a foamier state, thereby causing greater backscattering. The appearance of these materials, as well as the visible two-phase distribution shown in the photographs, can be observed in Figure 12, which compares samples immediately after synthesis and those stored for 24 h at 6 °C. Subsequently, an analysis was conducted to check the influence of mixing on the stability of the obtained material. Storage conditions and low-temperature “seasoning” have been shown to improve stability in similar formulations, facilitating better air bubble retention and phase uniformity [42]. Figure 11 (backscattering) and Figure 13 (transmittance) present individual scans from the multiscan for samples immediately after synthesis, after mixing, and then 24 h after mixing to check if the material returned to its original distribution post-mixing. Regarding the material not containing Tween, the material both after synthesis and after mixing exhibited the same spectral behavior, indicating the stability of the emulsion and homogeneity of the phases. However, 24 h after mixing, we see that the structure was more homogeneous, and the hydrophilic part of the substance may have collected in the upper layer, which had virtually no ability to scatter light because it did not contain air bubbles. For the graphs showing the backscattering of samples containing Tween, immediately after synthesis, we observe that all samples in the upper part exhibited a phase capable of greater scattering. After mixing, a homogeneous structure was observed for materials with a lower Tween content, while samples with 33% and 50% Tween exhibited quick separation even after mixing, and the high backscattering indicated the aeration of the sample post-mixing and a greater number of air bubbles stabilized by the addition of Tween. After 24 h, we observed the lowest backscattering values compared to previous measurements, indicating the effect of seasoning on the sample, where air bubbles may have been “escaping” from the sample. This could enhance the stabilization of the sample, i.e., seasoning it for an appropriate time at a low temperature. This allows us to conclude that in the process of obtaining the material, not only is the selection of appropriate mixture components important but also proper seasoning of the sample after preparation is crucial for achieving a material with the desired properties.

The final, but one of the most significant studies concerning the semi-solid materials, was the rheological study. Based on the obtained results, we observe that the addition of Tween caused an increase in the modulus of elasticity. This suggests that the addition of Tween may have led to a stiffer structure and, consequently, a more stable one [43]. These results provide information about the potential application of the prepared materials in applications that require structural stability, while the loss modulus indicates how the material responds to dynamic loads. Its high values may suggest that the obtained materials are less resistant to deformation due to dynamic forces; however, this is significant for us in terms of their potential applications.

The findings of this study highlight the potential of bilayer foamed oleogels as innovative carriers of active ingredients in cosmetic and pharmaceutical applications. Their improved rheological properties, wettability, and stability make them particularly suitable for formulations requiring controlled release and enhanced bioavailability. Additionally, the combination of Ecogel™ and Tween 80 contributes to the lightweight texture and cooling effect, further broadening their applicability to sensitive skincare and advanced therapeutic formulations, such as in wound healing or hydration systems.

## 4. Materials and Methods

### 4.1. Materials

In the oleogel formulations, the main components were a 2% gelatin solution, glycerin, Tween 80, and Ecogel™. The gelatin (extracted from bovine skin, Type B, powder), glycerin (colorless, viscous liquid, ACS reagent, ≥99.5%), and Tween 80 (Polysorbate 80, d = 1.07 g/mL) were bought from Sigma Aldrich (Saint Louis, MO, USA). Ecogel™ is a natural, biodegradable thickener and emulsion stabilizer, which was purchased from Lucas Meyer Cosmetics (Massy, France). The INCI designation of Ecogel™ is Lysolecithin (and) Sclerotium Gum (and) Xanthan Gum (and) Pullulan. All reagents were used as received, without further purification.

### 4.2. Synthesis of Oleogels

A 2% gelatin solution was prepared at 50 °C under constant stirring. Pre-measured amounts of glycerin, Ecogel™, and Tween 80 were added sequentially, followed by homogenization (8500 rpm, 5 min) using a Unidrive X1000 CAT homogenizer. The compositions of the formulations are shown in Table 1, with sample images presented in Figure 13. The prepared samples were immediately subjected to characterization.

After synthesis, the samples were immediately subjected to various investigations to verify their physicochemical properties. The main focus was on characterizing their stability, sorptive properties, wettability, morphology, chemical structure, and rheology.

### 4.3. Structural Characterization of Oleogels by FT-IR Spectroscopy

The presence of functional groups in the oleogels’ structure was verified by FT-IR (Fourier transform infrared) spectroscopy. FT-IR spectra were recorded using a Thermo Scientific Nicolett iS5 (Waltham, MA, USA) spectrometer equipped with a diamond ATR attachment (4000–500 cm^−1^, 32 scans, 0.4 cm^−1^ resolution). All spectra were recorded at room temperature.

### 4.4. Morphological Characterization of Oleogels via Optical Microscopy

The morphology of the oleogels was examined using a BRESSER Researcher Trino 40–1000× optical microscope (Bresser, Rhede, Germany) equipped with a Moticam 3.0 MP camera and Motic Image Plus 2.0 ML software (Motic, San Antonio, TX, USA) for image capture. Before imaging, the samples were thoroughly mixed with a glass dipstick to ensure structural homogeneity and uniform component distribution. Air bubbles, inherent to the foaming process, were not removed as they were considered part of the structural analysis. Representative images of the microstructure were obtained under these conditions.

### 4.5. Wettability Analysis of Oleogels

Wettability tests were conducted on both the homogenized mixture and the foam collected from the sample surface before mixing. The analysis used a nonpolar liquid (diiodomethane, δ = 50.80 mN/m) and a polar liquid (double-distilled water, Millipore, δ = 72.30 mN/m). Contact angle measurements were performed in triplicate using the Drop Shape Analyzer Kruss DSA100M (A. KRÜSS Optronic GmbH, Hamburg, Germany). The methodology for calculating surface free energy and its dispersive and polar components is detailed in a previous study [44].

### 4.6. Sorption Properties of Oleogels via Relative Humidity Measurements

The sorption ability of the oleogels was assessed by monitoring changes in relative humidity (RH) in a hermetically sealed Duran glass bottle containing the test sample. Inside the bottle, two Petri dishes were placed: one with 1.0 g of the test sample and the other with distilled, doubly deionized water. RH measurements were recorded every 5 min for 30 min, along with the temperature inside the bottle. The absolute humidity for each sample was calculated using a formula described in our previous study [44].

The relative humidity values were determined by means of the thermo/hydrometer Elmetron PWT-401 (Elmetron GP, Zabrze, Poland).

### 4.7. Evaluation of Oleogels’ Stability via Multiscan System

To evaluate the long-term utility of oleogels, their stability was investigated using the Multiscan MS-20 system (DataPhysics Instruments GmbH, Filderstadt, Germany). Three measurements were performed for each sample. First, oleogels were analyzed immediately after synthesis through continuous measurement for 30 min. Second, the samples were mixed and tested again using single scanning. Finally, mixed samples were analyzed after 24 h of storage at 6 °C to simulate refrigeration conditions commonly used for cosmetics and pharmaceuticals to extend shelf life. The storage at 6 °C allowed for assessing potential structural and physicochemical changes due to cooling, a critical consideration in real-world storage. This choice of temperature is further supported by common production processes, where oleogels are heated during synthesis and stabilized through cooling. Exemplary images of samples directly after synthesis and following 24 h of storage in a laboratory fridge are shown in Figure 12.

This study allowed us to verify whether there was stratification of the sample over time and separation of the different phases, crucial in terms of its application potential.

### 4.8. Characterization of Oleogels’ Rheological Properties

The rheological properties of the obtained oleogels were studied using a Malvern Bohlin Gemini II rheometer (Malvern Panalytical Ltd., Cambridge, UK) equipped with a sample temperature monitoring system using Peltier technology and Bohlin R6.51.0.3 software (Malvern Panalytical Ltd., Cambridge, UK). The measurement temperature was set at 25 ± 1 °C. A plate cone with a diameter of 40 mm and an angle of 4° was used, with a gap of 1000 µm.

For each developed oleogel, at least three measurements of rheological properties were performed. In the publication, we present the measurement results where the curves were closest to the mathematically defined average of all measurement results. The storage modulus (G′) and loss modulus (G″) of the samples were determined in the linear viscoelastic region (LVR) using a frequency sweep test (from 0 to 10 Hz).

### 4.9. Statistical Analysis

Statistical analyses were conducted to evaluate the significance of differences between experimental groups and the effect of Tween concentrations on the measured parameters. All data are presented as mean values with standard deviations (SDs), calculated based on three independent repetitions (*n* = 3).

For data involving two factors, namely, sample type (MIX vs FOAM) and Tween concentration (0%, 5%, 10%, 15%), a two-way analysis of variance (two-way ANOVA) was applied. This analysis assessed the main effects of both factors and their interactions. Subsequently, detailed comparisons between individual Tween concentrations within a specific sample type (MIX or FOAM) were performed using a one-way analysis of variance (one-way ANOVA) and Tukey’s post-hoc test to identify pairs of concentrations with statistically significant differences. Statistical significance was indicated on the graphs using asterisks (* *p* < 0.05, ** *p* < 0.01, *** *p* < 0.001). The significance level was set at *p* < 0.05.

For optical measurements, stability, FT-IR, and rheology, representative results are presented. These measurements were based on structural, dynamic, and continuous characteristics that demonstrated high reproducibility and consistency among samples of the same type. The results were selected from three repetitions and are considered representative of the analyzed systems, providing a reliable reflection of the properties of the studied samples.

## 5. Conclusions

The presented studies and their discussion allow for the formulation of several key conclusions:

1. The addition of Tween 80 intensified bands related to carbonyl, methylene, and ester groups, indicating changes in the chemical structure of the oleogels.
2. The presence of Tween 80 resulted in larger air bubbles and higher contact angles, suggesting increased porosity in the foam structure.
3. Samples with higher Tween concentrations showed the highest contact angles for water and greater dispersive energy, confirming their hydrophobic nature and affinity for non-polar substances like skin lipids.
4. Moisture analysis indicated that Tween promotes water release from oleogels. A multiscan analysis confirmed a two-phase, foamy structure and demonstrated that storage helps stabilize the materials by allowing air bubbles to gradually escape.
5. Higher Tween concentrations increased rigidity but reduced resistance to dynamic loads, suggesting improved stability but potential susceptibility to deformation.

In summary, Tween 80 significantly affects the physicochemical properties of oleogels, enhancing stability and hydrophobicity, which may be advantageous for cosmetic applications, especially for interactions with non-polar substances such as skin lipids. The results confirm that bilayer foamed oleogels represent a promising platform for developing multifunctional dermal formulations, offering unique benefits for both cosmetic and pharmaceutical industries. Their advanced properties provide opportunities for innovative product designs addressing market demands for effective, user-friendly, and sustainable formulations.

## Figures and Tables

**Table 1:** Compositions of the prepared oleogels.

	0_Tween	5_Tween	10_Tween	15_Tween
Ecogel™, mass fraction	6/30	6/30	6/30	6/30
Glycerin, mass fraction	30/30	30/30	30/30	30/30
2% gelatin solution, mass fraction	15/30	15/30	15/30	15/30
Polysorbate (vs. glycerin), %	+0%	+17%	+33%	+50%

## Data Availability

The data presented in this study are available on request from the corresponding authors.
